# The functional identification and evaluation of endophytic bacteria sourced from the roots of tolerant *Achyranthes bidentata* to overcome monoculture problems of *Rehmannia glutinosa*

**DOI:** 10.3389/fmicb.2024.1399406

**Published:** 2024-07-16

**Authors:** Chunli Zeng, Yazhou Liu, Bianhong Zhang, Chenjing Zhang, Niu Li, Leshan Ji, Chaojie Lan, Bin Qin, Yuncheng Yang, Juanying Wang, Ting Chen, Changxun Fang, Wenxiong Lin

**Affiliations:** ^1^Key Laboratory of Crop Ecology and Molecular Physiology, Fujian Agriculture and Forestry University, Fuzhou, Fujian, China; ^2^Fujian Provincial Key Laboratory of Agroecological Processing and Safety Monitoring, College of JunCao Science and Ecology, Fujian Agriculture and Forestry University, Fuzhou, Fujian, China; ^3^College of Life Sciences, Fujian Agriculture and Forestry University, Fuzhou, Fujian, China; ^4^College of Agriculture, Fujian Agriculture and Forestry University, Fuzhou, Fujian, China; ^5^College of Life Science, Guizhou University, Guiyang, Guizhou, China

**Keywords:** continuous monocultural obstacles, endophytic bacteria, plant growth-promoting, biological control, synthetic microbial consortium

## Abstract

The isolation and identification of plant growth-promoting endophytic bacteria (PGPEB) from *Achyranthes bidentata* roots have profound theoretical and practical implications in ecological agriculture, particularly as bio-inoculants to address challenges associated with continuous monoculture. Our research revealed a significant increase in the abundance of these beneficial bacteria in *A. bidentata* rhizosphere soil under prolonged monoculture conditions, as shown by bioinformatics analysis. Subsequently, we isolated 563 strains of endophytic bacteria from *A. bidentata* roots. Functional characterization highlighted diverse plant growth-promoting traits among these bacteria, including the secretion of indole-3-acetic acid (IAA) ranging from 68.01 to 73.25 mg/L, phosphorus and potassium solubilization capacities, and antagonistic activity against pathogenic fungi (21.54%−50.81%). Through 16S rDNA sequencing, we identified nine strains exhibiting biocontrol and growth-promoting potential. Introduction of a synthetic microbial consortium (SMC) in pot experiments significantly increased root biomass by 48.19% in *A. bidentata* and 27.01% in replanted *Rehmannia glutinosa*. These findings provide innovative insights and strategies for addressing continuous cropping challenges, highlighting the practical promise of PGPEB from *A. bidentata* in ecological agriculture to overcome replanting obstacles for non-host plants like *R. glutinosa*, thereby promoting robust growth in medicinal plants.

## Introduction

In modern agricultural practices, the limitations of arable land resources and the food demands for an increasing human population have driven an increase in intensive cultivation and long-term planting areas of single crops. It has been proved that the problems from consecutive monoculture could inevitably lead to reduced growth and development of crops as well as increased incidence of soil-borne diseases and higher yield penalties, which may entail substantial economic losses and compromise the sustainability of agriculture. Such is the case with Chinese medicinal plants, especially those with tuberous roots, that are continuously grown in the same field for years, often resulting in decreased pH value, soil pathogen enrichment and nutrient sequestration, and consequent reduction in yield and quality of crop in the cropping system. The aforementioned issue significantly hampers the sustainable development of Traditional Chinese Medicine (TCM) in China (Wu and Lin, 2020). Hence, it is imperative to gain a profound understanding of the underlying mechanisms behind continuous cropping obstacles and explore the effective control measures that have garnered substantial research attention.

The well-known traditional Chinese medicine *R. glutinosa*, belonging to the Scrophulariaceae family, is rich in various pharmaceutically active compounds such as iridoids, amino acids, and inorganic ions (Zhang et al., 2008). It is primarily cultivated in its geo-authentic source, Jiaozuo city, Henan Province, located in the central plains of China. However, continuous cropping has resulted in a significant decline in both yield and quality of *R. glutinosa* due to the outbreak of root rot pathogens (Wu et al., 2018; Li Q. et al., 2023). The successful eradication of soil memory resulting from continuous cultivation of *R. glutinosa* necessitates a rotation cycle exceeding 15 years, as well as the excessive use of chemical fertilizers (Yang et al., 2011). Consequently, this relocation process significantly diminishes both the yields and quality of geo-authentic areas for *R. glutinosa* medicinal plants. Exploring sustainable pathways is an effective strategy for alleviating replanting problems in *R. glutinosa* and enhancing crop productivity. Our previous studies have demonstrated that *A. bidentata*, originating from the same geographic source as *R. glutinosa*, exhibits tolerance to continuous cultivation and can grow normally after previous cropping of *R. glutinosa*. Furthermore, intercropping with *A. bidentata* has been found to effectively mitigate the replanting issues associated with *R. glutinosa* (Wang et al., 2019; Liu et al., 2022). The medicinal plant *A. bidentata*, belonging to the Amaranthaceae family, exhibits a unique characteristic of increasing yield and quality over successive years, unlike other medicinal plants that face significant obstacles due to continuous cropping (Li et al., 2010; Wang et al., 2019). Previous studies have demonstrated that *Fusarium oxysporum* derived from three rhizocompartments (rhizosphere, rhizoplane, and endosphere) of *A. bidentata* under a single continuous planting system did not attain dominance.

In fact, the abundance of plant growth-promoting endophytic bacteria (PGPEB) from the root of *A. bidentata* increases with continuous cropping years, particularly *Pseudomonas* and *Bacillus* species (Wang et al., 2021). PGPEB thrive within plants and enhance plant growth under both normal and challenging conditions by priming plant defenses, improving nutrient uptake, and modulating the synthesis of growth- and stress-related phytohormones (Qin et al., 2018; Borah et al., 2019; Carrión et al., 2019; Lau et al., 2020). Consequently, we hypothesize that the roots of *A. bidentata* could recruit rhizosphere-associated plant growth-promoting bacteria to adapt to continuous cropping environments.

To investigate the potential of PGPEB from *A. bidentata* in promoting its growth and alleviating the continuous cropping problem of *R. glutinosa*, this study aimed to isolate and culture endophytic bacteria from *A. bidentata* roots tolerant to replant disease, screen and identify PGPEB based on their plant growth-promoting and antagonistic traits, and ultimately develop a promising biofertilizer to mitigate replanting obstacles for *R. glutinosa*. This research will provide a technical pathway for exploring ecological approaches to prevent and control monoculture-related challenges, effectively safeguarding local geo-authentic sources while enhancing the yield and quality of TCM.

## Materials and methods

### Materials

Seeds of the *A. bidentata* variety “Hetaowen” were provided by Cuihong Lu from the Wenxian Institute of Agricultural Sciences, while the *R. glutinosa* variety “Jinjiu” was preserved by the Institute of Agroecology at Fujian Agriculture and Forestry University (FAFU), located in Fujian, China. The soil sample originated from a geo-authentic source in Jiaozuo City, Henan Province, China, with a composition containing 2.16 g/kg total nitrogen, 0.92 g/kg total phosphorus, and 0.99 g/kg total potassium, of which 184.33 mg/kg, 292.55 mg/kg, and 121.86 mg/kg were available, respectively. The pathogenic fungi *F. oxysporum* and Chromobacterium violaceum CV026 were preserved by the Institute of Agroecology at FAFU for further study; it is worth noting that CV026 does not possess sensitivity toward N-acyl-homoserine lactones (AHLs) auto-inducers, which are commonly associated with quorum-sensing bacteria (QSB). However, CV026 can be utilized as an AHL biosensor to detect QSB activity (Li et al., 2020).

### Methods

#### Bioinformatic analysis of plant-beneficial traits of endophytic bacteria from *A. bidentata*

In Jiaozuo city (113°22′E, 35°6′N), *A. bidentata* was sown during the final 10 days of June and harvested by mid-November within the same year. Following harvest, wheat cultivation took place from December until June of the subsequent year. Our previous study (Wang et al., 2021) provided a long-term (10 years) dataset comprising four root samples for *A. bidentata* root endophytic bacteria, obtained through continuous cropping: i.e., newly planted (A1Y), three-year consecutive monoculture (A3Y), five-year consecutive monoculture (A5Y), and ten-year consecutive monoculture (A10Y). In this study, we calculated the relative abundance and diversity in each sample by clustering reads from 16S rDNA amplicon sequencing into OTUs at 97% sequence similarities using USEARCH (Edgar, 2013). The resulting OTUs were aligned against the SILVA database to analyze alpha diversity indices such as Observed OTUs, Chao1, ACE, Shannon, and Simpson index with QIIME2 v1.9.1 according to literature (Bolyen et al., 2019). Community phylogenetic diversity indices including phylogenetic diversity (PD), phylogenetic species variability (PSV), phylogenetic species richness (PSR), and phylogenetic species evenness (PSE) were calculated using the R package picante based on literature (Kembel et al., 2010). Our focus was mainly on new results generated by potential functions of endophytic bacteria of *A. bidentata* based on microbial taxonomy and plant-associated functional traits, such as biocontrol capacity, PGP behavior, and stress resistance, found through the plant-beneficial bacteria (PBB) database (Li P. F. et al., 2023).

#### Isolation and purification of endophytic bacteria

The root samples of *A. bidentata* were collected from Jiaozuo city in November 2021. The collected root samples were thoroughly rinsed with running tap water to remove soil and subsequently washed with sterile water before processing. Surface sterilization of the samples was conducted as follows: roots were washed twice with 75% ethanol for 5 min each, followed by treatment with 4% sodium hypochlorite for 20 min, and finally washed three times with sterile water. To assess the effectiveness of surface sterilization, 0.1 ml of the last rinse water was plated onto LB agar medium.

The surface-disinfected roots were dissected into small fragments using a sterile scalpel and transferred to a sterile mortar. They were then crushed in 20 mL of sterile phosphate buffer saline (PBS). From this, 1.0 mL of the suspension was sequentially diluted until reaching a dilution factor of 10–3. A 0.1 mL aliquot from the dilution was spread onto LB agar medium, NA medium, and PISA medium (with 50 μg mL^−1^ natamycin) respectively. The plates were subsequently incubated at 37°C for 48 h (Ahmad et al., 2008). After the incubation period, morphologically distinct bacterial colonies on the plates were selectively picked and streaked repeatedly to obtain pure bacterial isolates. All purified bacterial isolates were stored at 4°C.

#### Evaluate the bacterial endophytes for growth-promoting traits

##### Phosphate solubilization

Each strain was cultured overnight and transferred onto Pikovskaya's agar medium at 28°C for 5 days to assess their phosphate solubilization ability (Boubekri et al., 2021). The formation of a clear zone around the colony, resulting from tricalcium phosphate utilization, was used as an indicator of phosphate solubilizers. The Phosphate Solubilization Index was calculated by measuring both the colony diameter and halo zone diameter using the following formula: Phosphate Solubilization Index = D/d = Diameter of clearance zone/Diameter of growth (Paul and Sinha, 2017).

##### Potassium solubilizing

Each strain was cultured overnight and then transferred onto Alexandrov's agar medium at 28°C for 5 days. The presence of a clear zone surrounding the colony was used as an indicator for identifying potassium solubilizers. The Potassium Solubilization Index (PSI) was calculated by measuring both the diameter of the halo zone and the colony, using the formula PSI = D/d = Diameter of clearance zone/Diameter of growth (Joe et al., 2018).

##### Siderophore production

The production of siderophores was qualitatively assessed following the method described by Schwyn and Neilands (1987). The strain was cultured overnight and inoculated with 2 μL onto CAS agar medium at a temperature of 28°C for a duration of 3 days. Siderophore production was confirmed by the presence of an orange halo surrounding the bacterial colonies on the medium. The Siderophore Production Index was calculated using the formula Siderophore Production Index = D/d = Diameter of zone of clearance/Diameter of growth (Ghazy and El-Nahrawy, 2021).

##### Produce IAA

Endophytic bacterial isolates were inoculated into nutrient broth media containing 0.2% (v/v) L-tryptophan and incubated at 28°C for 5 days with vigorous shaking. Controls consisted of 0.2% (v/v) L-tryptophan without bacterial inoculation. At the end of the incubation periods, the bacterial cultures were centrifuged at 12,000 rpm for 10 min at 4°C. 0.1 mL of the supernatant was mixed with one drop of orthophosphoric acid and 0.1 mL of Salkowski's reagent (300 mL concentrated sulfuric acid, 500 mL distilled water, 15 mL 0.5 M FeCl_3_). Development of a pink color indicated IAA production after 30 min (Mahgoub et al., 2021). To analyze the amount of IAA produced by each microalgal isolate, absorbance was measured at 535 nm using a spectrophotometer (Rushabh et al., 2020). The concentration of IAA was then calculated based on the standard curve (y = 0.0029x + 0.0384, R^2^ = 0.9988). The standard curve was generated by measuring the absorbance of IAA standards at known concentrations (10 mg/L, 12.5 mg/L, 25 mg/L, 50 mg/L, and 100 mg/L), plotting the absorbance values against the corresponding concentrations, and fitting a linear regression equation to the data.

##### Quorum sensing

The biosensor strain CV026 was used to streak with 2 μL strain which cultured overnight on the LB plates at 28°C for 48 h. The production of purple pigmentation by CV026 indicated that the co-cultured bacteria could produce QS signal molecules; these were identified as QS bacteria.

#### *In vitro* antagonistic activity

The antagonistic activity of bacterial isolates was assessed using the dual culture method on PDA medium *in vitro*. The fungal pathogen was centrifuged (10 min, 6,000 rpm) and its spores were then resuspended in sterile distilled water. Subsequently, the spore suspension was incubated at the center of a fresh PDA medium plate (90 mm in diameter). Approximately 2.5 cm away from the plug, a purified bacterial isolate (OD_600_ = 0.6) was symmetrically inoculated at four sites around the mycelial plug. Control group plates were only inoculated with each fungal phytopathogen. Dual culture plates and control plates were incubated at 28°C for up to 3 days. The percentage of inhibition was calculated using the formula (Swain et al., 2021) Inhibition (%) = (R1 – R2)/R1 × 100, where R1 represents the diameter of control plate and R2 represents the diameter of dual culture plate. The experiment was repeated three times with three independent replications.

#### Genotypic characterization and identification

The isolated bacteria were cultured overnight in LB liquid medium. The DNA of these strains was extracted using the E.Z.N.A Bacterial DNA Kit (Omega D3350-01), following the instruction manual for specific operational methods. Proper DNA quality for downstream procedures was assessed by 1% (w/v) agarose gel electrophoresis in TAE buffer. Extracted DNA was quantified using a NanoDrop spectrophotometer (Thermo Scientific™).

The extracted DNA samples were subjected to PCR amplification of the 16S rDNA sequence using the universal primers 27F (5′-AGAGTTT-G-ATCCTGGCTCAG-3′) and 1492R (5′-GGTTACCTTGTTACGACTT-3′) (Dos Santos et al., 2019). The PCR reaction mixture consisted of 25 μL of 2x TransStart^®^ GoldPfu PCR SuperMix, 2 μL Primer 27F (10 μM), 2 μL primer 1492R (10 μM), and 8 ng of DNA template, with a final volume of 50 μL made up with ultra-pure water. The thermal cycling was performed on an Applied Biosystems™ 2720 Thermal Cycler under the following conditions: initial denaturation at 94°C for 5 min, followed by a total of 35 cycles consisting of denaturation at 94°C for 30 s, annealing at 55°C for 30 s, extension at 72°C for 1.5 min, and a final extension step at 72°C for 10 min. Aliquots containing 10 μL of each reaction were analyzed on a 1% (w/v) agarose gel in TAE buffer. Subsequently, Tsingke Biotech conducted sequencing analysis on the obtained PCR products. The resulting sequences were assembled, edited, and aligned using DNAMAN and MEGA X software before being compared to those in the GenBank database through Basic Local Alignment Search Tool (https://blast.ncbi.nlm.nih.gov/Blast.cgi) to determine their homology with closely related organisms. Finally, in this study, the isolated bacteria were classified to species level based on information from the closest microbes.

#### *In vivo* inoculation pot experiments

The pot experiment was conducted in 2022 at the experimental field of the Institute of Agroecology, Fujian Agriculture and Forestry University. *A. bidentata* seeds were sown into pots (25 cm in height, 22 cm in diameter) filled with 4 kg soil sampled from the upper soil layer (5–20 cm) of fields at the Jiaozuo city experiment station. After germination, only one plant was retained per pot. The pot experiment consisted of two treatments and eight replicates: (i) newly planted *A. bidentata* plants inoculated with distilled water only (NPA) and (ii) newly planted *A. bidentata* plants inoculated with a mixed bacterial suspension (NPAB). The experiment commenced in June 2022, and the bacterial suspension was applied to each pot once a week starting on day 30 after thinning out the *A. bidentata* plants, repeated six times in total.

Finally, in November 2022, *A. bidentata* was harvested. Subsequently, an aliquot of 200 μL from each of the nine selected strains was individually incubated with 100 mL of LB medium and left overnight at a temperature of 30°C under constant agitation at 200 rpm. The bacteria were then collected through centrifugation and resuspended in sterile water. Each strain was adjusted to an OD_600_ value of 0.8 and mixed in equal proportions. For each pot, a total volume of 10 mL from the bacterial suspension mixture was inoculated using the same method for preparing the *R. glutinosa* pot experiment.

**Figure 1:**
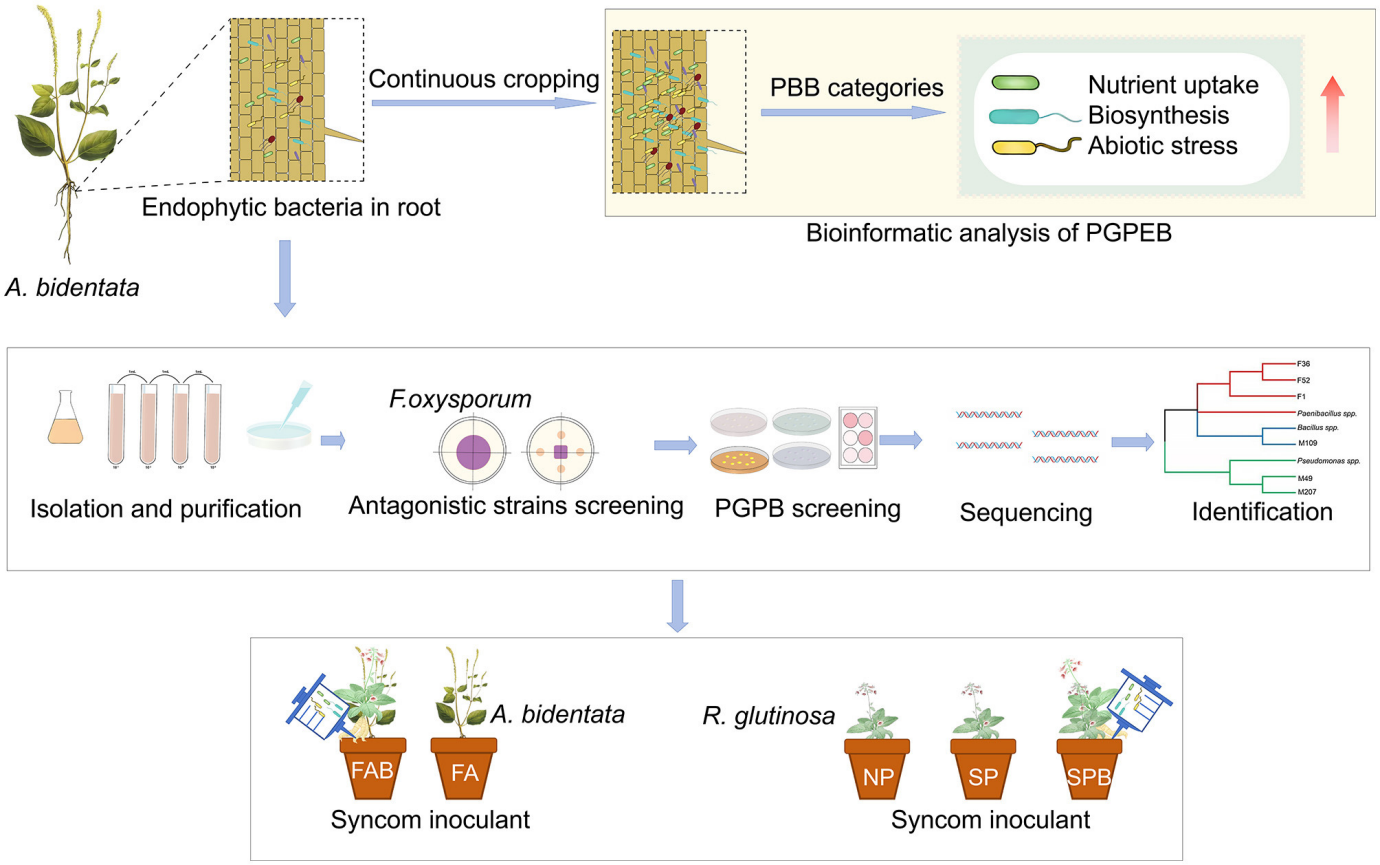
Experiment design. SMC: the synthetic microbial consortia; OSMC: the optimizing synthetic microbial consortia; NPA: newly planted *A. bidentata* were inoculated with distilled water only; NPAB: newly planted *A. bidentata* were inoculated with the bacterial suspension mixed; NPR: newly planted *R. glutinosa* were inoculated with distilled water only; CMR: continuous monocultural *R. glutinosa* was inoculated with distilled water only; CMRB: continuous monocultural *R. glutinosa* was inoculated with the bacterial suspension mixed.

The tubers of *R. glutinosa* were surface disinfected in a 0.3% carbendazim solution [N-(benzimidazlyl-2) methyl carbamate] for 5 min, followed by three rinses with distilled water. Subsequently, the tubers were planted into pots (25 cm in height and 22 cm in diameter) filled with soil samples collected from the upper layer (5–20 cm depth) of fields at the Jiaozuo City experiment station. Two types of soils were selected for the experiment: newly planted soil that had never been previously used to grow *R. glutinosa* and 1-year monoculture soil that had been cultivated with *R. glutinosa* for 1 year. The pot experiment involving *R. glutinosa* was designed with three treatments and eight replicates: (i) Newly planted *R. glutinosa* inoculated with distilled water only (NPR); (ii) Continuous monocultural *R. glutinosa* inoculated with distilled water only (CMR); and (iii) Continuous monocultural *R. glutinosa* inoculated with a mixed bacterial suspension (CMRB). In April 2022, two *R. glutinosa* plants were placed in each pot and, after 45 days of planting, they were inoculated once a week with the mixed bacterial suspension for a total of eight repetitions. Finally, in November 2022, *R. glutinosa* was harvested. The fertilization and other field management practices remained consistent across all treatments. The experimental design was shown in Figure 1.

#### Physicochemical properties of rhizosphere soil

The soil samples were collected using the method described by Wang et al. (2019). Upon harvesting the tuberous roots of *R. glutinosa*, the bulk soil was shaken off and ~2 mm of soil attached to the roots was collected. The samples obtained from each treatment were mixed and then divided into two parts for analysis. One part of the samples was stored at −80°C for DNA extraction, while the other part was used for analyzing the physicochemical properties of the soil. The total nitrogen (TN), phosphorus (TP), and potassium (TK) content were determined by initially digesting the soil using a H_2_SO_4_-H_2_O_2_ solution. Subsequently, TN was quantified using the Kjeldahl method, while TP and available phosphorus (AP) extracted with a NaHCO_3_ solution were analyzed using a colorimetric method. TK and available potassium (AK), extracted with ammonium acetate, were measured using flame atomic absorption spectrometry. The calculation of available nitrogen (AN) involved converting available nitrogen into ammonia.

#### Root activity of *R. glutinosa*

The root activity of *R. glutinosa* was assessed using the Triphenyl tetrazolium chloride (TTC) method (Bajracharya, 2004) on the 30th, 60th, 90th, 120th, 150th, and 180th days after planting.

#### Quantitative PCR of specific microbial taxa in different soils

The total DNA was extracted with BioFast Soil Genomic DNA Extraction Kit (BioFlux, Hangzhou, China) following the instructions. The DNA concentration was measured using a Nanodrop 2000C Spectrophotometer (Thermo Fisher Scientific, United States). A quantitative PCR assay was performed using the CFX96 Real-Time system (Bio-RDA, U.S.A) to determine the abundance of *Pseudomonas* (Ps-F 5′-TTA GCTCCACCTCGCGGC-3′/Ps-R 5′-GGTCTGAGAGGATGATCAGT-3′), *Bacillus* (BacF 5′-GGGAAACCGGGGCTAATACCGGAT-3′/1378 5′-CGGTGTGTACAAGGCCCGGGAACG-3′), and *Fusarium oxysporum* (ITS1F 5′-CTTGGTCATTTAGAGGAAGTAA-3′/AFP308R 5′-GGAATTAACGCGAGTCCCAA-3′). Each qPCR reaction mixture (20 μl) consisted of 2 × Green qPCR MasterMix 10 μL, forward and reverse primers 0.4 μL each, sterile water 8.2 μL, DNA template 1 μL.

### Statistical analysis

Graphs were plotted using Microsoft Excel 2019, Origin 2022b, GraphPad Prism 9.5, R 4.2.9 and SPSS 22.0 statistical software for significance analysis (Duncan's multiple range tests, α = 0.05); molecular biology identification was performed using DNAMAN for sequence splicing.

## Results

### Changes in endophytic bacteria diversity and function potentials of *A. bidentata* under consecutive monoculture

**Figure 2:**
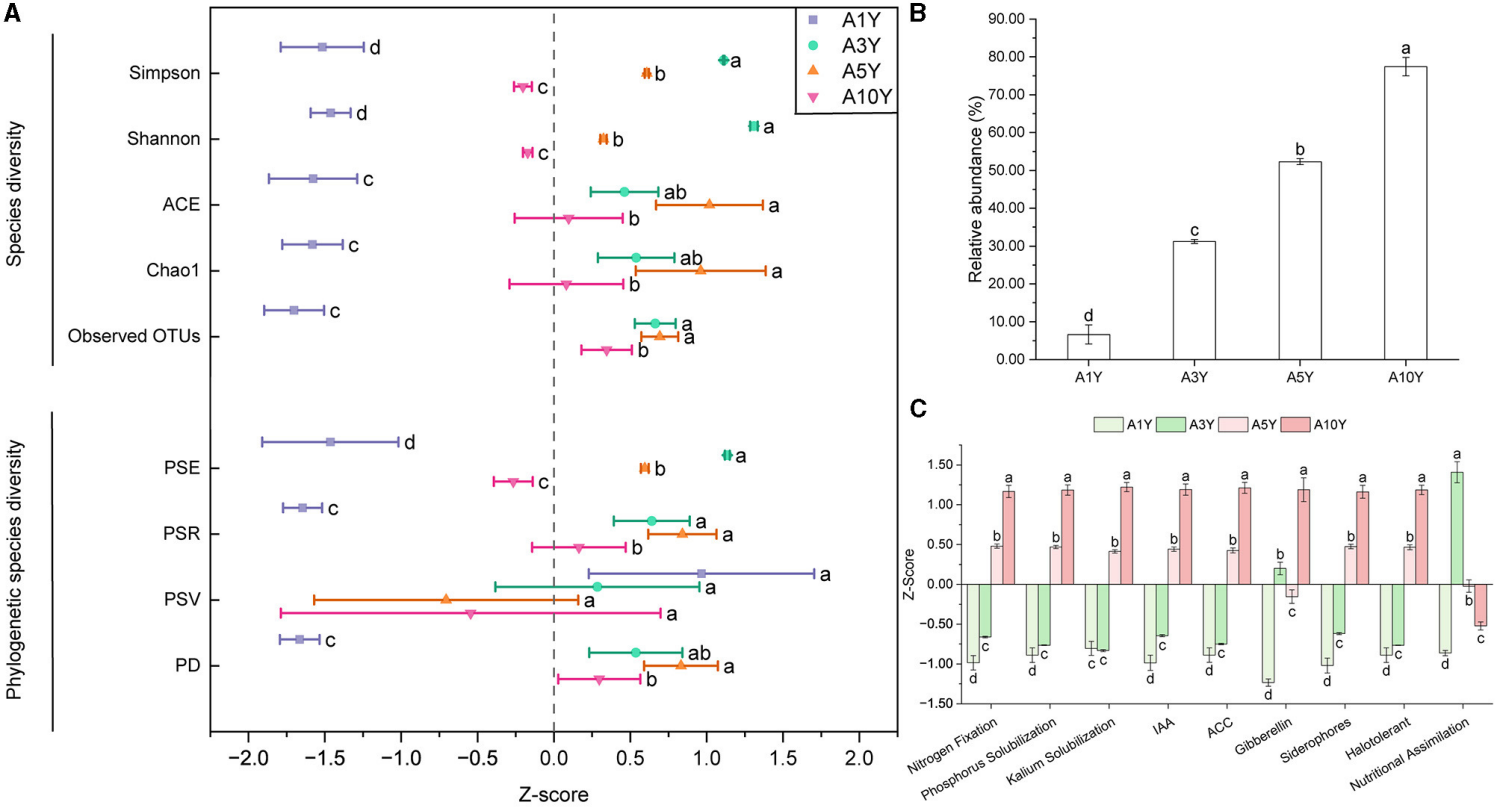
Changes of endophytic bacteria from *A. bidentata* root under different monocultural years. **(A)** Species and phylogenetic diversity of endophytic bacteria from *A. bidentata* root; **(B)** Changes in relative abundance of endophytic probiotics of *A. bidentata* under continuous cropping; **(C)** Prediction of functional changes of endophytic probiotics of *A. bidentata* under continuous cropping; different small letters indicate significant differences between treatments (*p* < 0.05, *n* = 3). PD, phylogenetic diversity; PSV, Phylogenetic species variability; PSR, Phylogenetic species richness; PSE, Phylogenetic species evenness; A1Y, A3Y, A5Y, A10Y represent *A. bidentata* under 1-year, 3-year, 5-year, and 10-year consecutive monoculture, respectively.

The analysis of Observed OTUs, Chao1, ACE, Shannon, and Simpson indices in the root of *A. bidentata* subjected to long-term monocultivation (A3Y, A5Y and A10Y) revealed that replanted *A. bidentata* exhibited more increased diversity in endophytic bacterial communities compared to newly planted ones (Figure 2A). Moreover, this trend was consistent when considering phylogenetic diversity indices such as PD, PSR, PSV, and PSE (Figure 2A). Based on the PBB database, this study further predicted the potential functions of endophytic bacteria. As depicted in Figure 2B, there was a substantial year-on-year increase in the abundance of beneficial endophytic bacterial community, with a significant rise of 77.44% observed at the tenth year of continuous cropping (A10Y). The classification analysis of beneficial endophytic bacteria (Figure 2C) revealed that successive cropping of *A. bidentata* led to a remarkable enhancement in the abundance of endophytic bacteria exhibiting various functions including nutrient uptake (nitrogen fixation, phosphorus solubilization, potassium solubilization, nutritional assimilation), biosynthesis (IAA, ACC, gibberellin, siderophores), and response to abiotic stress conditions, particularly evident at the tenth year.

### Growth-promoting and biocontrol traits

**Figure 3:**
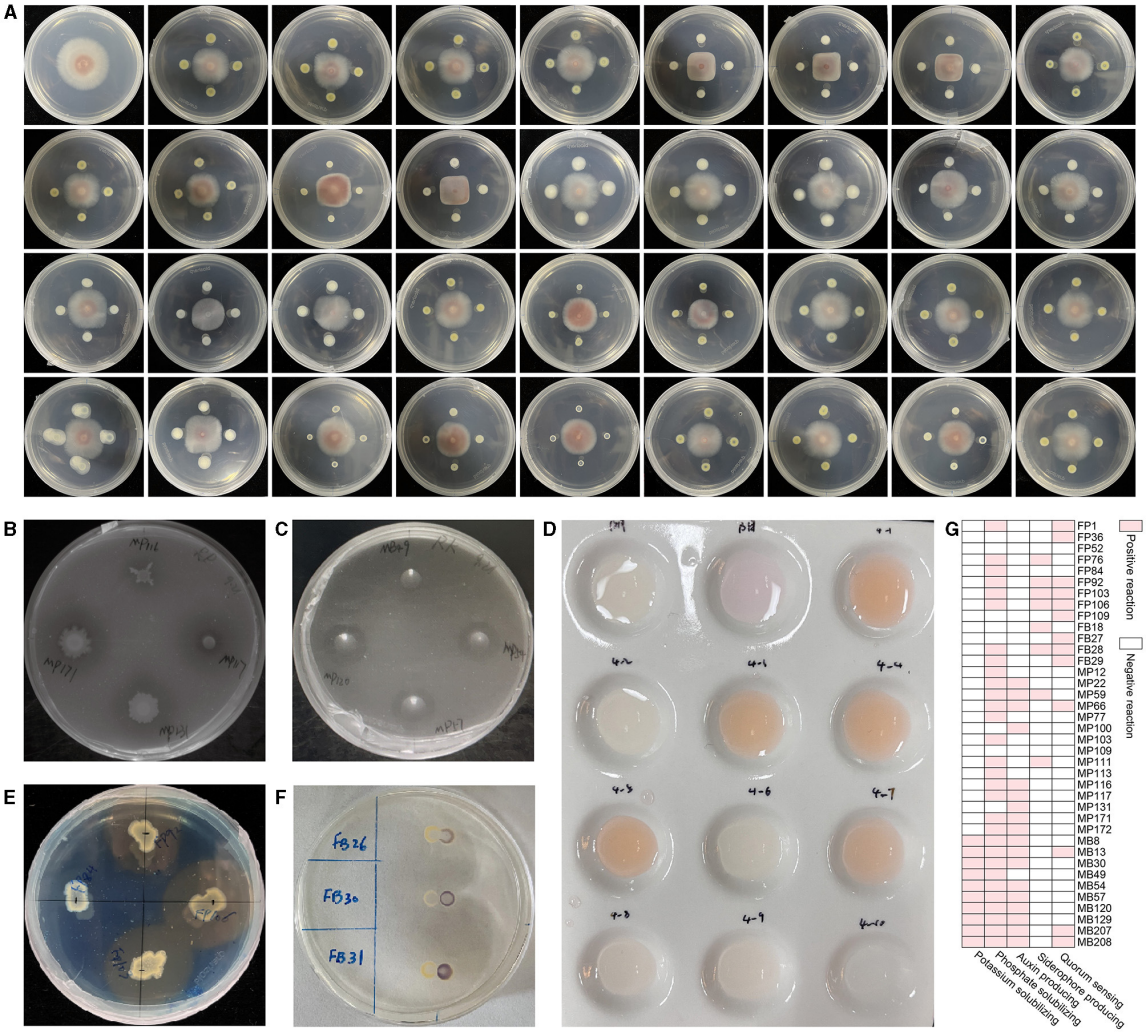
The qualitative effect of plant growth-promotion and antagonism of endophytic bacteria from *A. bidentata* root. **(A)** Antagonistic effect of total strains against the pathogen *F. oxysporum*. **(B)** Phosphorus solubilization, **(C)** potassium solubilization, **(D)** auxin secretion, **(E)** siderophore production, **(F)** quorum sensing, **(G)** total plant growth-promoting trait of strains.

In consideration of the ultimate objective of this experiment, which aims to address the issue of continuous cropping obstacles in *R. glutinosa* by introducing endophytic bacteria from *A. bidentata*, we have selected the ability to biocontrol *F. oxysporum* as the primary indicator for further strain identification. Through the dual culture method on PDA medium, we isolated 38 strains that exhibited inhibitory effects on the growth of *F. oxysporum* (Figure 3A). The antagonistic activity against pathogenic *F. oxysporum* ranged between 21.54% and 50.81% among these strains (Table 1).

**Table 1:** Inhibition of endophytic bacteria against pathogen.

**Strain**	**Inhibition (%)**	**Strain**	**Inhibition (%)**	**Strain**	**Inhibition (%)**	**Strain**	**Inhibition (%)**	**Strain**	**Inhibition (%)**
FP1	50.81 ± 0.02^a^	FP109	47.97 ± 0.01^a^	MP66	21.54 ± 0.03^j^	MP117	27.64 ± 0.05^efghij^	MB54	27.64 ± 0.01^efghij^
FP36	48.78 ± 0.00^a^	FB18	25.02 ± 0.01^hij^	MP77	30.89 ± 0.01^cdefgh^	MP131	27.64 ± 0.01^efghij^	MB57	30.89 ± 0.01^cdefgh^
FP52	49.59 ± 0.01^a^	FB27	30.89 ± 0.01^cdefgh^	MP100	29.27 ± 0.02^cdefghi^	MP171	35.37 ± 0.01^bcd^	MB120	29.27 ± 0.02^cdefghi^
FP76	32.11 ± 0.06^cdefg^	FB28	34.15 ± 0.03^bcde^	MP103	26.83 ± 0.00^fghij^	MP172	26.02 ± 0.03^ghij^	MB129	26.83 ± 0.03^fghij^
FP84	31.30 ± 0.03^cdefgh^	FB29	27.24 ± 0.05^fghij^	MP109	33.33 ± 0.01^bcdef^	MB8	23.17 ± 0.02^ij^	MB207	33.33 ± 0.06^bcdef^
FP92	31.30 ± 0.03^cdefgh^	MP12	28.86 ± 0.02^defghi^	MP111	27.24 ± 0.04^fghij^	MB13	25.20 ± 0.03^hij^	MB208	23.58 ± 0.06^ij^
FP103	35.77 ± 0.03^bc^	MP22	32.52 ± 0.06^bcdefg^	MP113	33.33 ± 0.01^bcdef^	MB30	21.54 ± 0.04^j^		
FP106	48.78+0.02^a^	MP59	39.02 ± 0.04^b^	MP116	32.93 ± 0.01^bcdef^	MB49	22.76 ± 0.06^ij^		

Different small letters indicate significant differences between treatments (*p* < 0.05, *n* = 3).

Among them, 30 strains exhibited positive results for phosphorus solubilization, while 10 strains showed positive results for potassium solubilization. The formation of a clear zone on agar containing insoluble phosphate or potassium by these strains indicated their ability to solubilize tricalcium phosphate and potassium, respectively (Figures 3B, C). To screen for the presence of indole-3-acetic acid (IAA), we employed the Salkowski reagent method. Interestingly, we observed that 18 strains exhibited secretion ability, with a more pronounced effect after 24 h (Figure 3D). Additionally, using the widely used CAS assay for siderophore detection, we identified eight strains as producers based on the development of an orange halo around bacterial colonies in CAS medium (Figure 3E). Furthermore, employing CV026 as a screening tool, we detected a total of 13 strains with quorum-sensing (QS) activity within just 24 h through a significant chromogenic response from the AHL reporter (Figure 3F).

### Identification of selected bacteria

Furthermore, the strains were validated by comparing their 16S rDNA nucleotide sequences to the NCBI database using the Basic Local Alignment Search Tool algorithm. Subsequently, these sequences were submitted to GenBank for accession numbers (Table 2). The results confirmed the identification of seven genera, namely *Acinetobacter* sp. (2), *Pantoea* sp. (13), *Raoultella* sp. (9), *Klebsiella* sp. (4), *Paenibacillus* sp. (3), *Bacillus* sp. (2), and *Pseudomonas* sp. (5).

**Table 2:** Molecular identification of strains based on 16SrDNA sequence.

**Strain**	**Bacterial name**	**Accession number**	**Identity (%) match**	**Reference organism**	**Access no. of ref. organism**
FP1	*Paenibacillus polymyxa*	PP449353	100%	*Paenibacillus polymyxa* strain KF-1	KT962922.2
FP76	*Pantoea sp*.	PP449354	97.4%	*Pantoea sp*. strain L-J-70	OP990834.1
FP103	*Acinetobacter nosocomialis*	PP449355	91.59%	*Acinetobacter nosocomialis* strain HBU72523	MW365222.1
MP171	*Raoultella ornithinolytica*	PP449356	99.38%	*Raoultella ornithinolytica* strain MG	CP017802.2
MB120	*Klebsiella sp*.	PP449357	99.49%	*Klebsiella sp*. MTJW-11	KM516019.1
MP109	*Bacillus sp*.	PP449358	88.54%	*Bacillus sp*. Pt2	JF900601.1
MP59	*Pseudomonas mosselii*	PP449359	99.59%	*Pseudomonas mosselii* strain yy161	MN177227.1
MP131	*Pseudomonas koreensis*	PP449360	99.58%	*Pseudomonas koreensis* strain D2	MG269614.1
MB129	*Klebsiella pneumoniae*	PP449361	99.90%	*Klebsiella pneumoniae* strain 84	MZ389292.1

### Assessment of potential plant growth-promoting bacteria of *A. bidentata*

**Figure 4:**
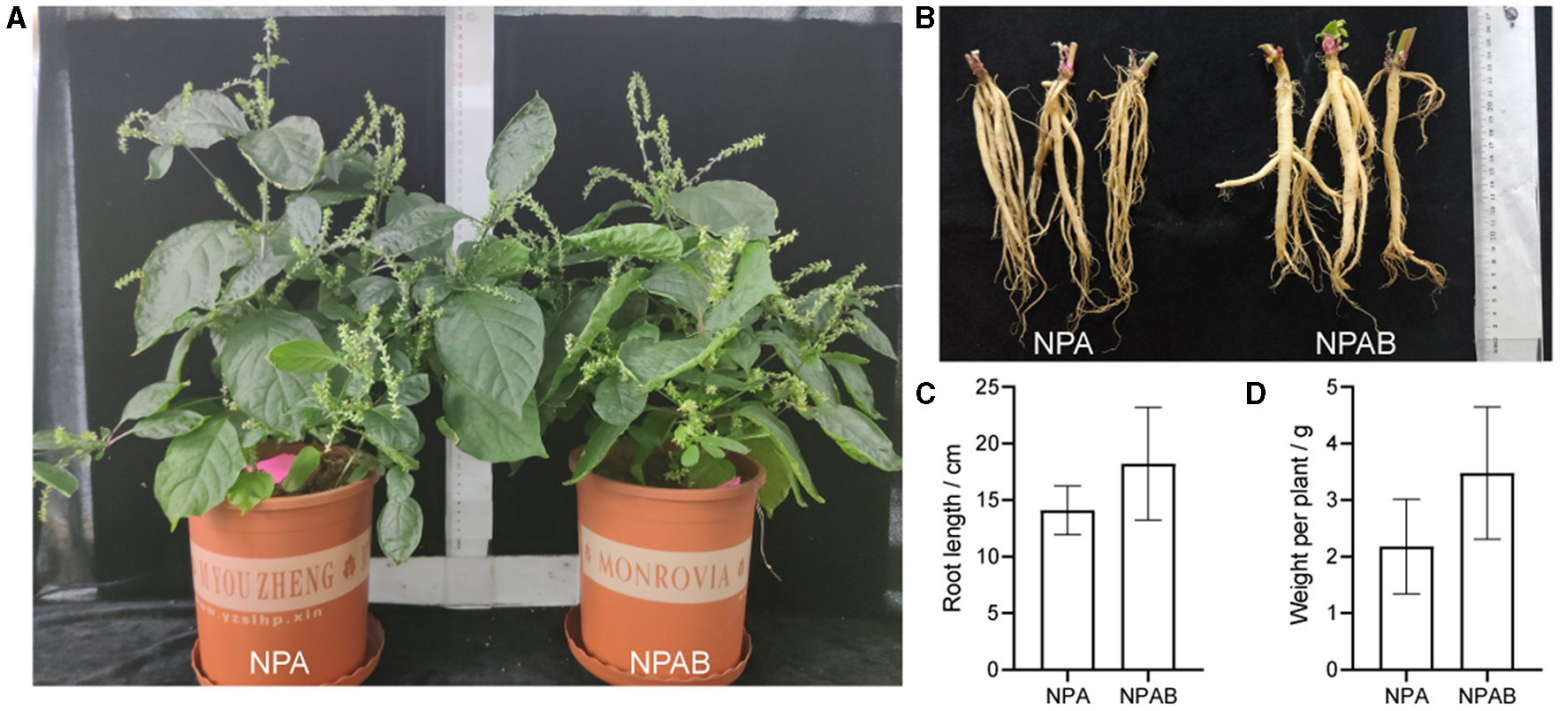
Effect of inoculation with the bacterial suspension mixed on the growth of *A. bidentata*. **(A, B)** Phenotypic changes of *A. bidentata* and its roots with inoculation. **(C, D)** Changes of root length and fresh weight of *A. bidentata* with inoculation. NPA, newly planted *A. bidentata* was inoculated with distilled water only; NPAB, newly planted *A. bidentata* was inoculated with the bacterial suspension mixed; different small letters indicate significant differences between treatments (*p* < 0.05, *n* = 3).

We selected nine strains for the application of biofertilizers based on microbial taxonomy and plant-associated functional traits, namely FP1, FP76, FP103, MP171, MB120, MP109, MP59, MP131, and MB129. The solubilization index and production index indicated that FP76 and FP103 exhibited the strongest phosphorus solubilizing activity among the strains tested. Additionally, MB120 and MB129 demonstrated the highest potassium solubilizing activity while FP76 and FP103 displayed superior siderophore-producing capabilities. Furthermore, using the IAA standard curve to determine concentration levels revealed that MP171 secreted the highest amount of auxin at 73.25 mg/L, followed by MP131 (73.16 mg/L), MB129 (69.64 mg/L), and MB120 (68.01 mg/L) (Table 3). Collectively, a total of nine strains from different genera exhibited a significant positive impact on growth-promoting functions such as phosphate and potassium solubilization, siderophore production, QS activity, IAA secretion, and antifungal activity. Therefore, these nine strains were combined into a synthetic microbial consortia (SMC) without mutual interference and introduced into the pot. The phenotypic growth parameters of *A. bidentata* in the pot are presented in Figure 4. These results demonstrate that inoculation can enhance the root fresh weight by 48.19% and length by 29.35% compared to the uninoculated treatment.

**Table 3:** Plant growth-promoting capacity of nine isolates.

**Strain**	**Quorum sensing**	**Phosphate solubilization index**	**Potassium solubilization index**	**Siderophore production index**	**Concentration of IAA (mg/L)**
FP1	+	1.50 ± 0.46d	-	-	-
FP76	-	3.27 ± 1.10a	-	2.31 ± 0.65	-
FP103	+	3.23 ± 1.09a	-	2.66 ± 0.87	-
MP59	-	-	-	-	48.75 ± 0.67d
MP109	+	1.65 ± 0.42d	-	-	-
MP131	-	2.42 ± 0.70b	-	-	73.16 ± 0.73a
MP171	-	1.95 ± 0.46c	-	-	73.25 ± 0.82a
MB120	-	1.52 ± 0.36d	1.87 ± 0.46	-	68.01 ± 0.76c
MB129	-	2.16 ± 0.71bc	1.94 ± 0.64	-	69.64 ± 0.59b

“+” Represents positive effect; “-” represents negative effect. Different small letters indicate significant differences between treatments (*p* < 0.05, *n* = 3).

### Evaluation of the application of SMC on the growth of *R. glutinosa*

**Figure 5:**
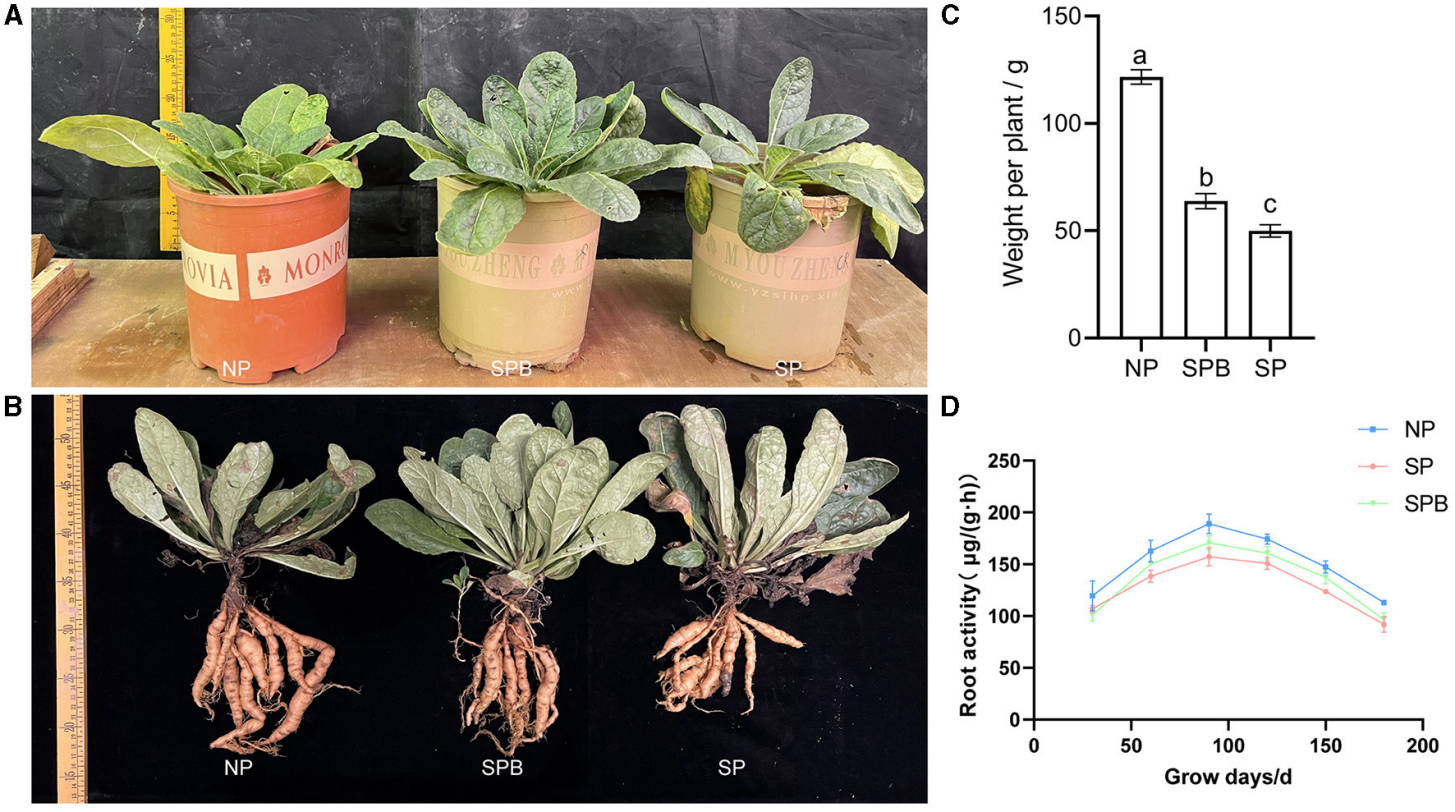
Effect of inoculation with SMC on the growth of replanting *R. glutinosa*. **(A, B)** Phenotypic of *R. glutinosa* and its roots with inoculation. **(C)** Fresh weight of *R. glutinosa* roots with inoculation. **(D)** Root activity of *R. glutinosa*. NPR, newly planted *R. glutinosa* was inoculated with distilled water only; CMR, continuously monocultural *R. glutinosa* was inoculated with distilled water only; CMRB, continuously monocultured *R. glutinosa* was inoculated with the bacterial suspension mixed; different small letters indicate significant differences between treatments (*p* < 0.05, *n* = 3).

As demonstrated by the aforementioned results, the application of SMC exhibited a growth-promoting effect on *A. bidentata*. However, it remained unclear whether SMC had similar biocontrol and growth-promoting effects on *R. glutinosa*. Therefore, we conducted another inoculation of SMC in the pots containing *R. glutinosa*, following the same procedure as described above. The findings indicated that, compared to replanted *R. glutinosa* without inoculant, the root fresh weight of replanted *R. glutinosa* inoculated with SMC significantly increased by 27.01%, although it did not surpass that of newly planted specimens (Figures 5A–C). Despite the negative impact of continuous planting on plant growth when compared to newly planted specimens, this adverse influence was considerably alleviated to some extent through SMC inoculation. The inoculant facilitated biomass accumulation in *R. glutinosa* and potentially influenced its physiology as well (Figure 5D). It is worth noting that, throughout all stages of growth and development, CMRB consistently displayed higher root activity than CMR.

### Effect and application of SMC on the rhizosphere of *R. glutinosa*

**Figure 6:**
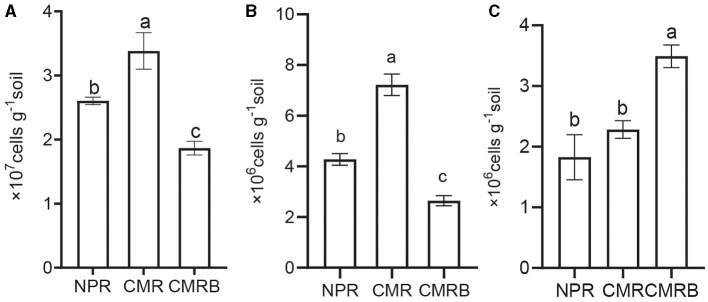
Quantitative PCR analysis of specific microbial taxa in different soil samples. **(A–C)** Quantitative PCR of the genus *Pseudomonas, Bacillus, F. oxysporum*; NPR, new planted *R. glutinosa* was inoculated with distilled water only; CMR, continuous monocultural *R. glutinosa* was inoculated with distilled water only; CMRB, continuous monocultural *R. glutinosa* was inoculated with the bacterial suspension mixed; different small letters indicate significant differences between treatments (*p* < 0.05, *n* = 3).

The physicochemical properties of the rhizosphere soil were assessed before and after planting (Table 4). The findings revealed that there were no significant disparities in the physical and chemical characteristics between CMR and CMRB soils, except for higher levels of AK and AP observed in the CMRB soil. Additionally, quantitative PCR results demonstrated a significantly greater abundance of *Bacillus* and *Pseudomonas* genera in CMRB compared to CMR, while *F. oxysporum* exhibited an opposite trend within the rhizosphere (Figure 6).

**Table 4:** Physicochemical properties of rhizosphere soil of *R. glutinosa* under different treatments.

**Stage**	**Treatment**	**TN (g/kg)**	**TP (g/kg)**	**TK (g/kg)**	**AN (mg/kg)**	**AP (mg/kg)**	**AK (mg/kg)**
Before planting	NPR	2.16 ± 0.11a	1.00 ± 0.04a	0.99 ± 0.06a	184.33 ± 1.25a	292.55 ± 3.94a	121.86 ± 6.14a
	CMR	2.47 ± 0.11a	1.19 ± 0.06a	0.70 ± 0.12a	265.33 ± 1.70a	257.59 ± 5.71a	166.3 ± 5.40a
	Harvest period	NPR	2.14 ± 0.10a	0.98 ± 0.02a	0.97 ± 0.07a	185.00 ± 1.73a	291.5 ± 1.73a	121.43 ± 6.54b
CMR		2.45 ± 0.21a	1.17 ± 0.05a	0.69 ± 0.18a	265.13 ± 0.58a	256.47 ± 4.65a	165.87 ± 6.92a
CMRB		2.45 ± 0.15a	1.18 ± 0.07a	0.70 ± 0.10a	265.17 ± 2.52a	257.09 ± 6.74a	166.19 ± 8.14a

TN, the content of total nitrogen; TP, the content of total phosphorus; TK, the content of potassium; AN, the content of available nitrogen; AP, the content of available phosphorus; AK, the content of available potassium; different small letters indicate significant differences between treatments (p <0.05, n = 3).

## Discussion

Plant-associated microbes play a crucial role in promoting plant growth, development, and stress tolerance (Rodriguez et al., 2019). Harnessing the potential benefits of these microbes offers a sustainable approach to enhancing crop productivity. In this study, we observed a significant increase in α-diversity of root endophytic bacteria from *A. bidentata* under consecutive cropping treatment. Furthermore, the endophytic microbiota exhibited various plant growth-promoting (PGP) traits such as indole-3-acetic acid secretion and phosphate and potassium solubilization, among others. By linking microbial taxonomy with their PGP traits based on PBB database, we propose that these probiotics may enhance host immunity and establish a protective layer during continuous cropping, thereby contributing to the improved yield and medicinal properties of *A. bidentata* after long-term monoculture. Indeed, our analysis demonstrates the PGP traits of these isolates obtained from diverse culturable bacterial endophytes in root samples. These findings provide novel evidence for the presence of culturable root microbiomes in *A. bidentata* and highlight their beneficial traits for plant growth. Overall, our results support the notion that microbiota and hosts co-evolve to mutually support growth and fitness (Dini-Andreote, 2020; Mukherjee et al., 2020; Abdelfattah et al., 2022).

Currently, a wide range of plant species can be colonized by endophytic bacteria, which have been isolated from various plant organs including roots, stems, leaves, seeds, fruits, and tubers (Dwibedi et al., 2022). Our research demonstrated that the root of *A. bidentata* harbored a diverse community of ~100 culturable endophytic bacteria, which played crucial roles in regulating plant growth and developmental processes through quorum sensing (QS), indole-3-acetic acid (IAA) synthesis, phosphate and potassium solubilization, as well as pathogen inhibition. Plate antagonistic experiments combined with DNA sequencing identified the antagonist isolates as *Acinetobacter sp., Pantoea sp., Raoultella ornithinolytica, Klebsiella sp., Paenibacillus sp., Bacillus sp., Pseudomonas mosselii, Pseudomonas koreensis*, and *Klebsiella pneumoniae*.

QS plays crucial roles in coordinating the functions of single-celled organisms, such as bacteria, within their family units (Dong et al., 2000). In this study, we discovered that FP1, FP103, and MP109 possess the ability to produce AHL signaling molecules in the QS system. It is well-established that certain gram-negative bacteria can respond to AHL signals and form biofilms (Medina-Martinez et al., 2007). Biofilms represent a communal lifestyle for bacteria, enabling them to exhibit enhanced social cooperation, resource acquisition, and survival capabilities compared to free-living cells (Davies, 2003). For instance, QS regulates both biofilm formation and antifungal activity of Serratia marcescens isolated from wheat stems (Liu et al., 2011). Additionally, biofilms aid microorganisms in evading host immunity and effectively colonizing their environment (Pang et al., 2009). Furthermore, endophytic bacteria contribute to crop growth and health by solubilizing potassium and phosphorus while producing siderophores (Dubey et al., 2020). Chen et al. (2015) demonstrated that *Acinetobacter calcoaceticus* Sasm_3_ exhibited the ability to enhance the growth of *Brassica napus* L, which aligns with the findings from our study on *Acinetobacter* sp. FP103. Previous studies have reported that *Pantoea* spp. can facilitate nutrient absorption and antagonize pathogens in *Blumeria graminis* (Rahman et al., 2018). Additionally, strains of *Pantoea* sp. were found to be effective against *F. oxysporum*, consistent with observations made in rice (Kouzai and Akimoto-Tomiyama, 2022). Similarly, *Paenibacillus* sp., like FP1, plays a crucial role in pathogen resistance and plant growth promotion through nitrogen fixation, phosphorus increase, and auxin production (Grady et al., 2016). Recent research work on plant growth-promoting bacteria has primarily focused on *Bacillus subtilis* (Errington and van der Aart, 2020). In our study, *Bacillus subtilis* MP109 exhibited significant antagonistic activity against pathogens. *Pseudomonas chlororaphis* (Liu et al., 2022) and *Pseudomonas psychrophile* (Ramírez-Bahena et al., 2015) are well-recognized as rhizosphere bacteria promoting plant growth.

*Pseudomonas mosselii* has been demonstrated to be an effective bacterium against the pathogenic fungi *Xanthomonas oryzae* and *Magnaporthe oryzae* (Yang et al., 2023). A previous study revealed that *Raoultella ornithinolytica* can inhibit the growth of *Plutella xylostella* and promote radish seedling growth (Guo, 2023). *Klebsiella* sp. MB120 and MB129 possess the ability to secrete indole acetic acid (IAA), which is consistent with other studies supporting our findings. For instance, *Klebsiella variicola* AY13, a bacteria known for producing IAA, has shown great potential in mitigating flooding stress and improving soybean plant growth (Kim et al., 2017). Additionally, similar to *Raoultella* sp. MP171, a previous study also demonstrated that *Raoultella ornithinolytica* inhibits *Plutella xylostella* growth while promoting radish seedling growth (Guo, 2023). These isolated strains clearly exhibit traits of promoting growth and biocontrol, as previously proven.

Compared to individual organisms, microbial cultures consisting of multiple species exhibit enhanced plant beneficial traits and resistance to environmental perturbations or invasions by other species as part of these microbial communities (Tsoi et al., 2018). The construction of SMC is a crucial approach for studying the function of the microbiome and its interaction with host plants (Vorholt et al., 2017). Our investigation demonstrated that SMC derived from culturable plant growth-promoting endophytic bacteria (PGPEB) isolated from *A. bidentata* have potential applications as biological inoculants for promoting plant growth. Consistent with our findings, reintroduction of isolated endophytes resulted in a significant increase in the length, biomass, and chlorophyll contents of *Cicer arietinum* (Mukherjee et al., 2020). Most of these studies have shown the potential growth promotion effects of endophytes isolated from the same plants (Santoyo et al., 2016).

Meanwhile, other studies have reported the growth-promoting effects of endophytic bacteria on non-host plants (Sessitsch et al., 2005; Ma et al., 2011). Our results from an *in-vivo* inoculation pot experiment, similar to *A. bidentata*, confirmed a reduction in continuous cropping diseases and an enhancement of *R. glutinosa* yield. Remarkably, we demonstrated that the potential for growth promotion by isolated endophytes can similarly exert a significant positive influence on different plants with habitat homogeneity. This research further substantiated that root activity of *R. glutinosa* was enhanced by the inoculant, activating insoluble mineral elements in soil to facilitate nutrient absorption availability. It has been proven that the application of SMC significantly enhances tomato resistance to the pathogen *F. oxysporum* (Zhou et al., 2022). Additionally, qPCR results showed that SMC inoculation increased beneficial bacteria such as *Pseudomonas* and *Bacillus* while decreasing pathogenic *F. oxysporum* amounts in the rhizosphere of *R. glutinosa*, resulting in reduced continuous cropping diseases and improved yield.

Originally, through bioinformatics analysis, we discovered a multitude of endophytic bacteria with PGP traits that were recruited during continuous planting of *A. bidentata*. Subsequently, we designed an experiment to investigate whether these beneficial endophytes could alleviate the challenges associated with continuous cropping of the non-host plant *R. glutinosa*. Our findings revealed that the isolated flora exhibited a higher tendency to colonize in the rhizosphere of the host plant. Recent studies have also demonstrated that endophytes are primarily sourced from the soil and rhizosphere (Edwards et al., 2015). Given that *A. bidentata* shares similar geo-authentic origins and habitat homogeneity with *R. glutinosa*, it is plausible to assume that they possess comparable rhizosphere microenvironments. Furthermore, we identified PGPEB as a biological inoculant capable of promoting growth in non-host plants while potentially exerting antagonistic effects against pathogens. However, one drawback was encountered: conditional pathogenic bacteria such as *Raoultella ornithinolytica* and *Klebsiella pneumoniae* were unsuitable for use as biofertilizers despite achieving our initial objective. Therefore, it is imperative for us to continuously optimize new and more viable solutions in order to develop effective biofertilizers for field applications. In conclusion, our work provides novel insights into mitigating or eliminating obstacles associated with continuous cropping for sustainable agriculture.

## Conclusions

In this study, it was evident that endophytic bacteria isolated from the root of *A. bidentata* exhibited promising potential in promoting plant growth. Furthermore, we demonstrated that these beneficial endophytic bacteria could alleviate the challenges associated with continuous cropping in non-host plant *R. glutinosa*. In other words, the PGPEB isolated from a medicinal plant *A. bidentata*, which possesses tolerance to continuous cropping, displayed biological activity toward *R. glutinosa*, a species facing severe replanting issues. Our future endeavor will focus on continuously exploring more rational approaches for constructing biofertilizer agents suitable for addressing replanting problems in Chinese medicinal plants.

## Data availability statement

The datasets presented in this study can be found in online repositories. The accession numbers for NCBI data are found with Table 2 of this article.

## Author contributions

CZe: Conceptualization, Data curation, Formal analysis, Methodology, Software, Validation, Writing – original draft, Writing – review & editing. BZ: Data curation, Methodology, Software, Supervision, Validation, Writing – original draft. CZh: Investigation, Methodology, Resources, Supervision, Writing – original draft. NL: Investigation, Methodology, Resources, Supervision, Validation, Writing – original draft. LJ: Investigation, Methodology, Resources, Supervision, Validation, Writing – original draft. CL: Data curation, Validation, Writing – original draft. BQ: Data curation, Methodology, Validation, Writing – original draft. YY: Investigation, Supervision, Validation, Writing – original draft. JW: Data curation, Resources, Writing – original draft. TC: Supervision, Validation, Writing – original draft. CF: Supervision, Validation, Writing – original draft. YL: Conceptualization, Formal analysis, Investigation, Methodology, Software, Supervision, Validation, Writing – original draft, Writing – review & editing. WL: Conceptualization, Investigation, Project administration, Supervision, Validation, Writing – original draft, Writing – review & editing.
